# The intractable problems with brain death and possible solutions

**DOI:** 10.1186/s13010-021-00107-9

**Published:** 2021-10-09

**Authors:** Ari R. Joffe, Gurpreet Khaira, Allan R. de Caen

**Affiliations:** 1 University of Alberta and Stollery Children’s Hospital, Division of Pediatric Critical Care, Edmonton, Alberta Canada; 2 University of Alberta, John Dossetor Health Ethics Center, 4-546 Edmonton Clinic Health Academy, 11405 112 Street, Edmonton, Alberta T6G 1C9 Canada

**Keywords:** Brain Death, Dead Donor Rule, Epistemology, Higher Brain Death, Metaphysics

## Abstract

Brain death has been accepted worldwide medically and legally as the biological state of death of the organism. Nevertheless, the literature has described persistent problems with this acceptance ever since brain death was described. Many of these problems are not widely known or properly understood by much of the medical community. Here we aim to clarify these issues, based on the two intractable problems in the brain death debates. First, the metaphysical problem: there is no reason that withstands critical scrutiny to believe that BD is the state of biological death of the human organism. Second, the epistemic problem: there is no way currently to diagnose the state of BD, the irreversible loss of all brain functions, using clinical tests and ancillary tests, given potential confounders to testing. We discuss these problems and their main objections and conclude that these problems are intractable in that there has been no acceptable solution offered other than bare assertions of an ‘operational definition’ of death. We present possible ways to move forward that accept both the metaphysical problem - that BD is not biological death of the human organism - and the epistemic problem - that as currently diagnosed, BD is a devastating neurological state where recovery of sentience is very unlikely, but not a confirmed state of irreversible loss of all [critical] brain functions. We argue that the best solution is to abandon the dead donor rule, thus allowing vital organ donation from patients currently diagnosed as BD, assuming appropriate changes are made to the consent process and to laws about killing.

Brain death (BD) has been accepted worldwide medically and legally as the biological state of death of the organism [1, 2]. Nevertheless, the literature has described persistent problems with this acceptance ever since BD was described [3–7]. Many of these problems are not known or properly understood by the medical community, as shown empirically [8–11]. Here we aim to clarify these sometimes confusing issues. Jumping ahead, the main goal is to clarify the metaphysical problem [i.e., why BD is not the biological state of death of the organism], and the epistemic problem [i.e., why the state of BD is not empirically verifiable at the bedside]. We will conclude with suggestions for moving forward in both accepting these problems and improving the practice of organ donation. Tables will give an outline of the problems, objections, and main replies discussed to help readers follow the flow of the paper. It is important to understand that the acceptance of BD is based on the theory that there is only one death per patient, and that this has occurred when the process of dying has ended and resulted in the irreversible state of BD [12–18]. There are two different ways to diagnose this singular state of death that occurs when there is absent brain function: tests for irreversible loss of all brain function while circulation continues (i.e., BD) or tests for irreversible absent circulation that can be known to have resulted in the irreversible loss of all brain function (i.e., cardiocirculatory death) [12–18].

## The Metaphysical Problem (Table 1 and 2)

### The BD hypothesis

BD is claimed to be the biological state of death of the human organism [12–18]. The explicit paradigm of death accepted in the medical literature is that death is a biological ontological state of an organism [12–18]. This means that death: i) is the event that marks the end of the biological physiological process of dying; ii) is a scientific reality, not merely a social contrivance nor a normative concept; and iii) applies to biological organisms.

**Table 1:** Timeline of influential authors that explicitly make the BD Hypothesis the standard medical and legal rationale for why BD has been accepted as a criterion for death of the organism

Reference; year	Significance	Statement	Page numbers
Korein; 1978 [19]	A main member of the medical consultants to the President’s Commission explaining, for a scientific forum on death, the standard concept of why brain death is equivalent to death	If the critical system, i.e., the brain, in a man is destroyed, the human organism is no longer in a state of minimal entropy production; its state will progressively become more disorganized by spontaneous irreversible fluctuations… irreversible cardiac arrest will inevitably follow regardless of maintenance of all resuscitative procedures… most often these final irreversible changes occur prior to 48 hours and even 24 hours after brain death.	26-27
Defining death: medical, legal and ethical issues in the determination of death; 1981 [12]	The President’s Commission that explicitly explained the concept of why brain death is equivalent to biological death	What was formerly a person is now a dead body and can be socially and legally treated as such. Although absence of breathing and heartbeat may often have been spoken of as "defining" death, review of history and of current medical and popular understanding makes clear that these were merely evidence for the disintegration of the organism as a whole, as discussed in Chapter Three.	58
		The first focuses on the integrated functioning of the body's major organ systems, while recognizing the centrality of the whole brain, since it is neither revivable nor replaceable. The other identifies the functioning of the whole brain as the hallmark of life because the brain is the regulator of the body's integration.	32
		On this view, death is that moment at which the body's physiological system ceases to constitute an integrated whole. Even if life continues in individual cells or organs, life of the organism as a whole requires complex integration, and without the latter, a person cannot properly be regarded as alive.	33
		This view gives the brain primacy not merely as the sponsor of consciousness (since even unconscious persons may be alive), but also as the complex organizer and regulator of bodily functions. (Indeed, the "regulatory" role of the brain in the organism can be understood in terms of thermodynamics and information theory). Only the brain can direct the entire organism. Artificial support for the heart and lungs, which is required only when the brain can no longer control them, cannot maintain the usual synchronized integration of the body.	34
		[Absent all brain functions] even with extraordinary medical care, [vital] functions cannot be sustained indefinitely – typically, no longer than several days.	35
The bifurcated legal standard for determining death: does it work; 1999 [20]	Alexander Capron, a drafter of the UDDA, explaining why brain death and cardiocirculatory criteria both meet the standard concept of death	…confirmed the existing concept of death as a phenomenon diagnosable by the two alternative methods… The circle of integrated functioning was broken, however it was assessed.	125
		…crystalizes the contemporary understanding of death because it illustrates how some of an organism’s vital parts remain functional even though the organism has died, namely, lost its ability to perform as an integrated whole because some essential element (typically, the brain) can no longer function and cannot be replaced.	126
Controversies in the determination of death: a white paper by the President’s Council on Bioethics; 2008 [13]	The President’s Council that explicitly re-addressed the standard concept of why brain death is equivalent to death	The neurological standard’s early defenders were not wrong to seek such a principle of wholeness. They may have been mistaken, however, in focusing on the loss of somatic integration as the critical sign that the organism is no longer a whole. They interpreted—plausibly but perhaps incorrectly— “an organism as a whole” to mean “an organism whose parts are working together in an integrated way.”	59-60
Interdisciplinary panel convened with support from the Health Resources and Services Administration Division of Transplantation; 2010 [21]	An international panel convened to address whether donation after cardiocirculatory death donors meet the standard concept of death	In its 1981 report Defining Death, the U.S. President’s Commission for the Study of Ethical Problems in Medicine and Biomedical and Behavioral Research provided the most frequently cited comprehensive analysis. Defining Death had three principal goals: 1) to provide a conceptual basis for the new medical practice of death determination using neurological tests…	963
American Academy of Neurology multisociety quality improvement intitiative, 2018 [22]	A summit “to address, and potentially correct, aspects of brain death determination within the purview of medical practice that may have contributed to these lawsuits”	After an extensive review, the Commission concluded that brain death should be endorsed as legal death, and produced the Uniform Determination of Death Act (UDDA)…	424
		Just as cardiopulmonary death is determined when there is irreversible loss of circulatory and respiratory function, brain death is defined by irreversible loss of consciousness and brainstem function leading to the inability to breathe independent of artificial support, and ultimately results in the demise and decay of all organ systems.	426

**Table 2:** The Metaphysical Problem with brain death, and outline of objections with their main replies

Argument	Objection	Replies
The Brain Death Hypothesis is false: we observe continued integration and homeostasis of the organism as a whole	Define death as loss of the ‘fundamental vital work’ of a living organism	-Not a scientific theory: the fundamental “drive” and unconscious “felt need” to continue to exist as an organism implies a ‘vital principle’ or ‘soul’. -Defined exclusively in terms of externally directed work: but, the goal of external work is to sustain the capacity for internal integrative unity [homeostasis]. -Does not serve the ad-hoc purpose for which it was constructed: the BD patient does demonstrate ‘openness to the world’, does ‘act upon the world to obtain what it needs’, and does demonstrate the ‘basic non-conscious felt need that drives the organism to act as it must, to obtain what it needs’.
	Integration is merely artificially maintained by the ventilator	-The ventilator is not causally sufficient for heartbeat or gas exchange: it simply blows air into the bronchial tree, and the integrated organism does all the rest. -The ventilator is causally necessary for the heartbeat and gas exchange: but so are many other functions that, when replaced, do not result in merely artificial integration. -Consciousness may be a ‘sui generis’ emergent property; however, it does not follow that, and is ad-hoc to assert that, some other [replaceable by technology] brain neurophysiologic functions are critical simply because the brain also generates consciousness.
	Define death as loss of personhood [higher-brain death]	-Still leaves the death of the integrated living biological organism as a whole to occur. -Another view is ‘Animalism’: personhood may only be a phase of our existence. -Unwelcome implications: the PVS patient is already dead and should be treated as such; I could never fall into a PVS; removing life-support from a patient in PVS would not kill one of us or violate the rights of any person; I was never a fetus; early abortion would not kill one of us or violate the rights of any person.
	Assert an operational definition of death as BD	-Dismisses long history of rigorous characterization of the biological death of an organism. -Misrepresents what philosophy is about: the goal of philosophy is to ensure clarity, logical consistency, and rational argumentation to arrive at reasoned conclusions. -Not science: void of any empirical or testable content. -Existential assertions are not socially constructed.
	Propose a homeostatic property cluster account of death	-The cases used to suggest current definitions conflict with our “best intuitions” are flawed. -Not much of a cluster: the same consciousness [‘personhood’], and biological organism [integration; all the rest of the properties] controversy. -Why accept that cluster: based on framing bias, and thus begs the question. -Based on raw intuitions: but, it is better to subject these to critical scrutiny. -Ignores the implication that the fetus is dead.

*BD* brain death, *PVS* irreversible permanent vegetative state

Necessary to understanding this is the concept of homeostasis: the ability to utilize external energy [by metabolism] to maintain a highly organized internal environment [extracellular fluid] fluctuating within acceptable limits [a necessary condition for all organismic functioning] [23, 24]. Homeostasis is the fight against entropy, the tendency towards chemical and thermal equilibrium according to the second law of thermodynamics [25]. With this concept, we can define a living organism as an integrated functioning organism as a whole, a localized pocket of anti-entropy achieved by maintaining internal homeostasis while resisting chemical and thermal equilibrium with the external environment [12, 14–16, 23–25].

Conversely, we can define death as the irreversible cessation of the integrated functioning of the organism as a whole, such that the organism no longer has the capacity to restore homeostasis and thereby resist entropy [12, 14–16, 23–25]. Death is a thermodynamic point of no return – entropy and disintegration take over.

The BD Hypothesis is that without the brain serving to integrate and unify the dynamic metabolic processes, the organism is no longer a unified whole that acts together to maintain homeostasis and resist entropy and disintegration [12, 14–16, 23, 24, 26]. In the state of BD, there is only a mere collection of parts, and not an organism. This is why the state of BD meets the concept/definition of death - that there is no longer an organism as a whole. In medicine and hence law, BD has been, and continues to be, accepted as death with this justification [4–7, 12, 14–16, 26–28]. In Table 1 we present a timeline of influential papers and authors that explicitly make this BD Hypothesis clear as the standard medical and legal rationale for why BD has been accepted as a criterion for death of the organism [12, 13, 19–22].

### The problem

The problem is that it is now known that BD is not the loss of integrated functioning of the organism as a whole [3–7, 13, 23, 24, 26–28]. This was empirically shown by D. Alan Shewmon in 1998, when he described cases of ‘chronic BD’ with ‘survival’ durations of 1 week for n=161, 2 weeks for n=67, 4 weeks for n=32, 2 months for n=15, and >6 months for n=7 [29]. The Kaplan-Meier survival curve looked similar to that for patients with terminal illnesses that were nevertheless alive. Since that time, more cases have been reported, particularly in pregnant women who had ‘chronic BD’ for weeks and months until a viable fetus was born [30–32]. The longest duration of BD was reported to be 20 years in a boy who suffered BD from meningitis at age 4 years, was sustained at home most of the time with nothing more than ventilation and enteral feeds, and whose heart stopped at age 24 years after which autopsy found no neural elements identifiable intracranially [i.e., he surely had whole-brain death] [33].

These cases demonstrated many signs of homeostasis including the following: “homeostasis of a countless variety of mutually interacting chemicals, macromolecules and physiological parameters, through the functions especially of liver, kidneys, cardiovascular and endocrine systems”, “elimination, detoxification and recycling of cellular wastes throughout the body”, energy balance, maintenance of body temperature, wound healing, fighting of infections (including development of a febrile response), “cardiovascular and hormonal stress responses to unanesthetized incision for organ retrieval”, successful gestation of a fetus, sexual maturation (i.e., puberty), proportional growth, “resuscitatability and stabilizability following cardiac arrest, and ability to bounce back from episodes of hypotension, aspiration, sepsis and other serious systemic setbacks”, “spontaneous improvement in general health… i.e., the gradual stabilizing of cardiovascular status so that initially required pressor drugs can be successfully withdrawn, the gradual return of gastrointestinal motility so that initially required parenteral fluids and nutrition can be successfully switched to the enteral route… ability to maintain fluid and electrolyte balance” with no or rare monitoring or adjustments in fluid and hormonal therapy [5]. In fact, cases demonstrated “the overall ability to survive with little medical intervention (although with much basic nursing care) in a nursing facility or even at home, after discharge from an intensive care unit” [5].

BD does not lead inevitably to disintegration of the organism nor to cardiac arrest. Integration is an emergent non-localizable property of a living organism that does not require an integrator nor a central organizing unit [34]. The brain is more an enhancer than an indispensable integrator of bodily functions [34]. This is also evident when one considers that the early human fetus, an integrated biological living organism in which the brain is forming but has not become active, is not an inanimate entity, nor a mere aggregate of living cells and tissues. This empirical refutation can be put more precisely as the following argument [23]:

Premise 1: H. The BD Hypothesis states that BD is perfectly correlated with the irreversible cessation of functioning of the organism as a whole.

Premise 2: HͻO. If H is true, then, we expect to observe irreversible cessation of functioning as a whole, and the entropic process should take over.

Premise 3: ~O. We often observe that homeostasis maintaining functions continue (i.e., integrated functioning of the organism as a whole).

Conclusion: ~H. The BD Hypothesis is false.

It might be argued that Premise 3 fails (i.e., it is not clear that homeostasis maintaining functions continue), as there exists no measuring scale for the ‘degree of integration’ of a complex system, and there is difficulty trying to conclude anything from comparing lists of somatic functions/dysfunctions. Shewmon has refuted these claims with the following arguments [5, 34]. First, Premise 3 succeeds even accepting that there exists no measuring scale for ‘degree of integration’ of a complex system.

Premise 1: Dying patients in intensive care units, with multiple organ dysfunction syndrome and a rapid downhill spiral, by virtue of being still alive, are necessarily on the ‘whole organism’ side of the hypothesized dividing line.

Premise 2: Many patients with BD in intensive care units are as stable as, and some are more stable than, such dying patients. BD patients can be at home, with no more support than a ventilator, tube feedings, a few medications, and good nursing care.

Premise 3: Such BD patients are also on the ‘whole organism’ side of the dividing line.

Conclusion: The organism during BD is not merely a bag of partially interacting subsystems, but rather is an integrated functioning organism as a whole.

Second, Premise 3 succeeds even accepting the difficulty of trying to conclude anything from comparing lists of somatic functions/dysfunctions [34, 35].

Premise 1: A functionally brain-disconnected patient on a ventilator in an intensive care unit (e.g., from high spinal-cord transection, or extreme Guillain Barre Syndrome) is a severely disabled organism functioning as a whole. This patient is not just a conscious head connected to an unintegrated collection of organs and tissues enclosed in a bag of skin.

Premise 2: The somatic effects of brain non-function are necessarily identical to those of brain disconnection [say, with the vagus nerve cut].

Premise 3: A patient with brain non-function is also a severely disabled organism functioning as a whole.

Conclusion: The organism during BD is an integrated functioning organism as a whole.

Although the standard reason for why BD was accepted as death, we acknowledge that not all have agreed with the BD hypothesis and its concept of death as loss of integration of the organism as a whole. In what follows, we consider all other widely offered alternative concepts of death and other objections to the BD hypothesis.

### Objections

#### Reject H: propose a new definition of death

The President’s Council on Bioethics suggested in 2008 that a living whole organism can be defined as one in which there remains “the persistence of the fundamental vital work of a living organism – the work of self-preservation, achieved through the organism’s need-driven commerce with the surrounding world ” [13]. This fundamental vital work was said to require three capacities: i) “Openness to the world, that is, receptivity to stimuli and signals from the surrounding environment”, ii) “The ability to act upon the world to obtain selectively what it needs”, and iii) “The basic [non-conscious] felt need that drives the organism to act as it must, to obtain what it needs and what its openness reveals to be available” [13].

This new teleological definition of life and death fails for several reasons. First, this is not a scientific biological theory. If being alive requires a fundamental ‘drive’ and non-conscious ‘felt need’ to continue to exist as an organism, this would mean that death is the departure of this animating or vital principle [i.e., soul] from the organism [36–38]. However, ‘vital principle’ and ‘soul’ are not scientific biological concepts. Second, wholeness is defined exclusively in terms of externally directed work. If internally directed work [e.g., self-development and self-maintenance, homeostasis] does not count, this would mean that the fetus, the patient with permanent vegetative state (PVS) and inability to breathe, and the totally locked-in patient are all dead organisms [36–39]. In fact, the goal of external work [the so-called fundamental vital work of self-preservation], is to sustain the capacity for internal integrative unity [maintenance of internal homeostasis] [23, 36]. Third, the new definition does not even serve the ad-hoc purpose for which it was constructed [36]. The BD patient does demonstrate “openness to the world” with receptivity to stimuli and signals: the patient will clot blood at and heal tracheostomy and gastrostomy tube incisions, may have withdrawal spinal reflexes, may fight off infections, and may have hypertension and tachycardia to organ retrieval. The BD patient does “act upon the world to obtain selectively what it needs”: the patient assimilates nutrients and electrolytes from fluids and feeds in the world, eliminates unneeded wastes in stool and urine to the world, and exchanges gases with the world in ventilated lungs. The BD patient does demonstrate the “basic [non-conscious] felt need that drives the organism to act as it must, to obtain what it needs”: the patient has basic drives to circulate blood with oxygen and nutrients to sustain its vital organs, to absorb needed nutrients and eliminate unneeded wastes from the bowel, to acquire needed oxygen from the lungs, all of these allowing for growth, puberty, and recovery from complications [24, 40].

#### Reject ~O: claim that the integration is artificially maintained

Some claim that the biological reality of death is masked by the intervention of mechanical ventilation. The President’s Commission in 1981 wrote that the ventilator “generated breathing, heartbeat, and the associated physical characteristics (e.g., warm, moist skin) of life… Respiration and circulation are, therefore, solely artifacts of mechanical ventilation [and] mask this loss of integration [p. 22-23]” [12]. The President’s Council in 2008 similarly wrote that “the apparent signs of life that remain- a beating heart, warm skin, and minimal, if any, signs of bodily decay – are a sort of mask… an artifact of technological intervention… The simulated ‘breathing’ that the ventilator makes possible is not, therefore, a vital sign… the exchange of gases that it effects is neither an achievement of the organism nor a sign of its genuine vitality [p.3, 63-64] ” [13].

This claim fails for several reasons. First, the ventilator is not causally sufficient for heartbeat or gas exchange. If one were to intubate and ventilate an actual corpse, this will not result in a heartbeat or gas exchange or any other sign of life. The ventilator simply blows air into the bronchial tree. The organism does all the rest: gas exchange and circulation are achievements of the integrated functioning of the organism as a whole [23, 41]. Second, the ventilator is causally necessary for the heartbeat and gas exchange, but so are many other functions. All these functions are together jointly sufficient to maintain the background conditions necessary for heartbeat and gas exchange [23, 26, 41]. Thus, there are two arbitrary and ad-hoc claims being made. One is to prioritize breathing above other equally necessary physiological functions. The organism can be supported or enabled by a pacemaker, dialysis, insulin, a vasopressor, a caregiver, and, of course, ventilation. All of these are instruments of life-support that can only work if there is still life present in the organism [26, 41]. The other is to claim that functioning must be natural in the irreversibly unconscious patient, but not in the conscious patient [40]. This would imply that the patient with PVS is dead the instant they require any form of life-support. This would also imply the fetus is dead, because it is entirely dependent on the mother’s body for life-support. Finally, we ask, would you have an open casket funeral or bury a BD patient while on a ventilator, with ongoing circulation? If not, isn’t this because they are not yet dead? [40].

Recently, Bernat has suggested an updated defense of the BD Hypothesis based upon a “deeper understanding of the organism as a whole [OaaW]” [16, 42]. The OaaW is “an antientropic entity [‘oppose the thermodynamic force towards increased entropy and disorganization’] with processes promoting increasing biological complexity, which results in an integrated wholeness through emergent properties [‘becoming a mereological whole that possesses a life status distinct from that of its derivative parts’]” [42]. This mereology “is uniquely characterized by finality, its derivative parts (organs and organelles) instrumentally serve the whole organism as the final end and benefactor” [42]. There are “differing biological levels of complexity”, and the “most macroscopic unifying and integrating emergent functions of each OaaW type [are] critical for that organism to be that kind of OaaW ” [42]. The human OaaW “has a more complex neurological structure permitting not only sentience and wakefulness, but also more exquisite emergent functions such as self-awareness, abstraction, sapience [to use reasons], and insight” [42]. These higher conscious functions are “sui generis (‘a kind of its own’)… qualitatively different from nonbrain functions because they are nonreducible [e.g. cannot transplant the brain; cannot be replaced by technology]” [42]. For this reason, the “neurological center [brain] is the primary and final… organismal integrator,” giving the human organism a “final emergent neurobiological structure” [42]. Bernat claims that the “neuro-emergent functions” of the neurological center include both homeostatic neurophysiologic functions [“like respiration and circulation”] and critical conscious [“wakefulness and capacity for self-awareness”] functions. So, “it is the neurological integrating centers seating the biological functions, not the biological functions per se, that defines the human OaaW”, and “it is the neurological center seating the conscious functions, not the conscious functions per se, that define the human OaaW” [42]. Since BD is the “irreversible loss of the human neurological center (the brain)”, it is death of the human OaaW [42]. This defense fails. First, even if consciousness is a “sui generis” function, it does not follow that the brain neurophysiologic functions should be included in the “final emergent neurobiological structure.” As argued above, many non-brain emergent functions are equally necessary to realize consciousness, and the brain neurophysiologic functions actually can be and are replaced by technology. This flaw is evident in Bernat’s claim that Spinal Cord functions are not included because their “integration appears distinct from the perceptual processing and mental content involved in the production of further sui generis neuro-emergent brain functions”, and “some functions of the spinal cord can be substituted or replaced by technology” [42]. Second, the account seems contrived to account for the “corporeal-conscious” [biological criteria vs. consciousness criteria] incompatible intuitions, instead of subjecting these intuitions to critical scrutiny. It is ad-hoc to include some brain neurophysiologic functions as critical simply because the brain also generates consciousness. It is also odd to claim that the brain is critical and final because it generates consciousness, but it is “not the conscious functions per se, that define the human OaaW.” Third, we agree with Shewmon when he writes that “it is not clear to me whether his most recent proposal of consciousness as the *sine qua non* critical function of the human ‘organism as a whole’ differs substantially from the mentalist rationale rejected in his previous writings or merely dresses it in the formulational garb of mereology and emergent functions” [43]. This higher brain death proposal is discussed next.

#### Reject H again: propose a personhood definition of death

Some suggest that death occurs when there is so-called ‘higher brain death’. Shemie has written that “one of the pivotal conceptual advances in human pathophysiology [is that] the non-functioning of the brain signals death of the person” [44]. He writes that “being dead can be defined as absent brain function with no biological potential in the brain to reinstate sufficient cell function required to achieve emergence to consciousness and self-awareness” [45]. He writes elsewhere that “the capacity for consciousness and self-awareness is uniquely synonymous with human life and personhood, and its absence is necessary and sufficient to identify that death has occurred… The capacity for consciousness and self-awareness is the only irreplaceable emergent phenomenon….” [46].

There are strong intuitions that motivate accepting this view. The transplant intuition is that, if you were to have a head or brain transplant, you would go with your head or brain, not stay with your remaining [perhaps supported and integrated] organism [37, 47–51]. The so-called remnant person problem is that, if you were to have “really gruesome guillotining” where you were beheaded and your brain sustained for some time prior to transplantation [or just sustained mid-transplant], you would go with your brain and not stay with your remaining organism, and you would be a [remnant] person during the time you were a brain alone even though you did not have a body [52]. These are not merely thought experiments, as head transplantation has been done in non-human animals and is planned in humans as well [53–56]. Considerations of rare types of conjoined twinning also motivate this view [57, 58]. Dicephalus describes twins fused below the neck where, intuitively, there are clearly two distinct persons that share a single biological organism; since the twins are not identical to each other, neither is identical to the biological organism. Cephalopagus describes twins with a single head and cerebrum with, intuitively, one distinct person with two possibly separable biological organisms below; since one person cannot be identical with two non-identical biological organisms, neither organism is identical with the person. The problem is that “it is impossible to maintain that dicephalus is two organisms while cephalopagus is only one” [57]. One either has to accept that there is only one organism in both forms of twinning, or two organisms in both forms of twinning. So, if in dicephalus there are, actually, two overlapping organisms [i.e., one person per biological organism], then in cephalopagus there are also, actually, two organisms [i.e., one person for two biological organisms], and *we* cannot be identical to the organism. Conversely, if in cephalopagus there is, actually, one organism [i.e., one person per biological organism], then in dicephalus there is also, actually, only one organism [i.e., two persons per biological organism], and we cannot be identical to the organism. This shows that our identity conditions are those of the functional brain [“the functional areas of the brain that are necessary and jointly sufficient for the capacity for consciousness”] [58].

Despite this initial intuitive appeal, there are problems. Personhood is not a biological nor a scientific concept [23]. The question of what kind of thing we essentially are is metaphysical. Thus, it is important to understand what the higher brain death concept is really saying - there are two deaths for each of us (death is not a univocal concept): the biological death of my human organism, and the death of *me* the human person. This would mean that *I* am essentially an entity with a capacity for consciousness, and not an organism; “death of the human organism will necessarily be *my* death only if *I* am an organism” [47]. There are several problems with this claim. First, there is still the death of the integrated living human organism as a whole (i.e., biological death) to occur in the BD body. The body is internally integrated to maintain biological homeostasis, as proved by actual BD bodies [i.e., “not on the same ontological level as an amputated limb”] [34]. In the remnant person case, the sustained head [*me*] is not a living organism, as this “requires a great deal more than a pump [blood must be renewed (bone marrow), cleansed (liver, kidney), supplied with nutrients and oxygen (digestive system, lungs), etc.]… [The head] has no internal regulation or integration [everything it needs for survival must be externally supplied] ” [58]. Second, personhood is not the only theory of what we essentially are. Some argue that *we* are the organism, a view called Animalism [6, 51, 59–62]. The claim is that personhood may be a phase of our existence as a human organism, similar to adolescence. To cloak the personhood concept under the mantle of science, thus granting it a social and epistemic authority, is misleading [23]. Third, there are unwelcome metaphysical implications of the higher brain death concept [47–49]. If consciousness is required for personhood, then the patient with PVS is already dead, and should be treated as such [i.e., buried, cremated, autopsied] despite breathing, moving, wakefulness, etc., because only my living ‘humanoid’ mere organism remains [37]. Indeed, *I* could never fall into a PVS [only my organism could]. Removing the feeding tube from a patient in PVS would not kill one of *us*, or violate the rights of any person. In addition, *I* was never a fetus; my organism was a fetus, but *I* began later on. Early abortion would thus not kill one of *us*, or violate the rights of any person. If self-awareness is required for personhood, then *I* was never a neonate; my organism was a neonate, but *I* began later on. Infanticide would not kill one of *us*, or violate the rights of any person. In addition, *I* could never have severe dementia, only my organism could.

Several authors have not understood these points, and thus made metaphysical mistakes when they advocate the higher brain death concept. For example, the claim that consciousness “entails a state of being awake *and* aware of self and environment [so that] a patient in a PVS may lack awareness but demonstrates arousal and cannot be considered deceased ” [18]. This claim includes two simple logical errors based on the fact that (a & b), as in (awake & aware), is not the same as (a V b), as in (awake or aware). First, the claim means that awareness must be present for consciousness, and thus in fact means that the PVS patient is to be considered dead. Second, the claim means that wakefulness must be present for consciousness, yet consciousness can occur during wakefulness or [REM] sleep, and thus may not require wakefulness at all. Another example is the claim that death of the person requires “no brainstem reflexes” [44–46]. However, brainstem reflexes are irrelevant to consciousness or personhood; the claim conflates ‘consciousness’ with ‘all brain functions’. Similarly, the claim that death of the person requires “no ability to breathe independently” [46]. However, apnea is irrelevant to consciousness or personhood; the claim again conflates ‘consciousness’ with ‘all brain functions’. Moreover, breathing is not an “irreplaceable function of the brain” [44–46]. Another example is making the analogy to decapitation, claiming that the BD individual has physiological decapitation, and is dead just as a decapitated individual is dead; this is sometimes supplemented with pictures of decapitation to ‘prove’ that BD is death [63, 64]. Shewmon has analyzed this claim, asking what is the essential aspect of hypothetical progressive decapitation that makes it ‘equivalent’ to death: i) non-neural non-vascular elements cut- no (this leads to surgical repair); ii) vascular elements cut- no (this only leads to death by bleeding); iii) neural elements cut- no (this often leads to rehabilitation); iv) complete severance- maybe (this certainly leads to death). The essential aspect of decapitation that is said to make it ‘equivalent’ to BD is having neural elements cut, and this is different from what may make decapitation death [65]. The analogy fails to explain why BD should be death, and is not an argument for the higher brain death concept.

#### Reject the need for a concept of death at all: assert an operational definition [brainstem death]

A recent international panel developing guidelines for the determination of death asserted that a concept [i.e., definition] of death is “an abstract, unprovable explanation of death, generally based on religious, spiritual, or philosophical beliefs” [17]. In addition, they assert that there is an accepted “biomedical operational definition of death”, that is, “death is the permanent loss of the capacity for consciousness and all brainstem functions” [17]. Some have suggested revising the Uniform Determination of Death act to stipulate this [e.g., “with the exception of hormonal function”] [66]. In effect, this is a claim for a brainstem death criterion for death.

This claim fails for several reasons. First, this dismisses a long history of rigorous scientific work in characterizing the concept of the biological death of an organism [36]. The scientific biological concept is of course not based on religious, spiritual, nor philosophical beliefs. The biological status of BD patients is not at all vague: they resist entropy and maintain homeostasis, and thus are not dead organisms [23, 24, 36, 41]. In fact, the loss of personhood concept *is* based on religious, spiritual, or philosophical beliefs. Second, this misrepresents what philosophy is about. Philosophy is a way to subject assertions to critical scrutiny, clarifying exactly what the assertion is saying, its implications, and thus its direct plausibility [67]. Surely it is not wise to discard this goal of ensuring clarity, logical consistency, and rational argumentation to arrive at reasoned conclusions. Third, this claim is no longer science at all; rather, the claim is “only authoritative assertion void of any empirical or testable content” [36]. The following argument demonstrates why this is so:

Premise 1: To operationalize a concept is to provide measurable observable criteria that coincide with that concept.

Premise 2: Operational criteria are meaningful only relative to some particular concept that the proposed criteria are intended to operationalize.

Premise 3: An operationalization of no concept whatsoever is meaningless.

Conclusion: The operational definition is not science, but is only an assertion of a tautology [i.e., the same as saying “the criterion BD is the criterion BD”]. In other words, this is only restating the criterion of brainstem death, and not stating a concept/definition of death that this criterion meets [26, 36].

Maybe the claim has been misinterpreted. The claim may be that the problem of a definition of death also applies to the definition of when life begins (i.e., abortion debates), and an operational definition is used in those debates. This claim is false. No one argues that the embryo and fetus are not alive, or are not living human organisms. Biologically, it is recognized that the embryo and fetus are alive. The question debated is whether these human organisms are due the same moral regard that more fully developed humans are owed [68]. Or, the claim may be that the operational definition of death is the best we have. This claim is also false. The best definition offered by science that we have for biological death is the loss of integration of the organism as a whole [12, 14, 23–28, 36]. And we have a good criterion for this also – when circulation stops irreversibly [12, 14, 26, 36, 69, 70]. Or, the claim may be that legally a patient is dead when diagnosed as dead according to accepted medical standards, i.e., when the doctor says you are dead, according to the operational definition [21]. This claim is also false and misleading. Medical professionals cannot just say anything; the correct claim should be that the patient is dead when the doctor says so, *because of why* the doctor says that [70]. Surely there must be an adequate justification for how the doctor has diagnosed death. Finally, the claim may be that when to call someone dead is a matter of social agreement or construction, asserting that everything in biology is on a continuum. This claim is also false. Existential assertions [e.g., “x is” or “x does not exist”] do not refer to phase sortals [i.e., “death is not a phase, but the end of all phases in the life of an organism”] or kind sortals [i.e., “death is not a sortal that distinguishes between kinds”, but refers to “an individual member of some biological natural kind that has died”] [71]. We do not merely arbitrarily decide or stipulate when to call someone dead – existential assertions are not continuous and are a judgment of fact [71].

#### Reject H again: propose a homeostatic property cluster account of death

Chiong has suggested that there may be no shared characteristic common to all dead things in virtue of which they are dead [72, 73]. Instead, there are only homeostatic property clusters, families of properties that tend to be nonaccidentally coinstantiated in natural biological kinds. Semantically, we can use a so-called “operational definition that helps us focus on the object of our inquiry, even if it does not reveal the underlying nature of the object [i.e., may appeal to merely accidental rather than indispensable characteristics]” [72]. In particular, in indeterminate borderline cases, where it is unclear whether something belongs to the relevant kind, we can perform “sharpening [of] the distinction [precisification] by introducing an artificially defined cutoff” [73]. In the case of death, the proposed cluster includes: consciousness, spontaneous vital functions [those necessary for the persistence of the other functions of the organism; regulated and maintained by activities that are internal to the organism], behavior [functional responsiveness] to environmental stimuli regardless of consciousness, integrated and coordinated functioning of multiple subsystems [organization complexity and coherence], ability to resist decay and putrefaction, capacity to reproduce, and capacity to grow via the assimilation of nutrients [72]. Consciousness, a property central to the cluster, is said to be sufficient for life even when present alone, and presence of one or several of the other properties, peripheral to the cluster, may not be sufficient for life [72, 73]. To motivate this cluster view, Chiong describes so-called counter-intuitive borderline cases: cardiopulmonary death - a patient suffering irreversible loss of circulation and respiration, and thus permanent loss of integrated functioning, even though with briefly retained consciousness, is dead; PVS: although lacking consciousness, has other classic signs of biological life [spontaneous breathing, sleep/wake cycles, brainstem reflexes], and so is not dead; Donation after Circulatory Death – permanent loss of circulation and breathing occurs even though “we know that many people retain some primitive brain functions [such as the gag reflex] for several minutes after their hearts stop beating [i.e., this does not approximate BD] ” [72, 73].

This proposal is interesting, but fails for several reasons. First, the cases used to show that current definitions conflict with our “best intuitions”, and thus to motivate the need for a cluster account are flawed. When circulation has *irreversibly* ceased there is no longer any “briefly retained consciousness”, nor is there “retain[ed] some primitive brain functions [such as the gag reflex]”. The PVS patient has ongoing integration, just as the BD patient does; of note, a PVS patient could lack many brainstem reflexes as long as only one continues, weakening the distinction between PVS and BD. Second, the cluster is, well, not much of a cluster. It sounds like only two properties: consciousness (‘personhood’) and integration (biological organism - all the other properties). This leads to comparing lists of (central and peripheral) integrative functions to determine who is dead, and as discussed above, the BD are not dead using this approach [5, 34]. Third, why accept that cluster? - “the distinction between ‘revision’ and ‘sharpening’ is unclear and seems to beg the question” [74]. In other words, framing bias [interests, values, and ontological assumptions] “bear on the selection of how significant certain properties in the cluster are to the classification” [74]. As Chiong admits, “we seem to be stuck appealing to our own intuitions about what strikes us as commonsensical, so it is difficult to be confident that we’re actually tracking distinctions out in the world rather than projecting our values” [75]. It is possible that one may tailor the standards to meet the purpose of facilitating organ procurement. This may be why some questionable claims were made by Chiong to defend the distinction between BD and PVS: spontaneous breathing is central to the cluster, with ventilation considered being “so dependent on external provision of such paradigmatically vital functions as breathing” [but, as discussed above, many other vital functions are also necessary to survive]; blink or cough are privileged responses [more central than fluid/electrolyte maintenance or breathing at a PaC02 of 80 mmHg]; and testing for hormone secretion and thermoregulation are “difficult and costly and might thereby delay [diagnosis]”, and are therefore not central properties [though simply observing urine output and temperature may suffice to determine their presence] [72, 73]. Fourth, Chiong only claims that it *may be* the case that there is no shared characteristic that defines when death occurs. It also may not be the case. The better response to our intuitions is to subject them to critical scrutiny, to clarify what exactly is being claimed and its implications, rather than to use the intuitions as raw data [67]. We already know a great deal about the physical processes involved in death [7]. The “evidence shows quite clearly that these patients’ status with respect to biological life is not at all vague” – the organism resists entropy and maintains homeostasis, and is therefore not dead [23, 24, 76]. Fifth, the implication for the fetus is ignored. The first trimester fetus does not have consciousness, blinking, coughing, or even breathing, and thus is biologically dead by the cluster account.

#### Similar metaphysical claims could be made about using circulatory criteria to diagnose death

This objection is the claim that the circulatory death criterion, the irreversible loss of circulation in the organism, is not perfectly correlated with the irreversible cessation of functioning of the organism as a whole. Some have supported this assertion with the claim that irreversible circulatory cessation does not lead to loss of all brainstem function. These claims are false. First, irreversible loss of circulation in the organism is in fact, according to currently accepted science, perfectly correlated with the irreversible cessation of functioning of the organism as a whole [69, 70]. Circulation of blood is a necessary condition to be an integrated organism as a whole, and with irreversible absent circulation the organism no longer has the capacity to restore homeostasis and thereby resist entropy. This is why the irreversible loss of circulation remains a *criterion* for the diagnosis of the state of irreversible loss of integration of the organism as a whole (i.e., biological death); irreversible BD does not indicate this loss, and thus is not a valid criterion for biological death. Second, when circulation has irreversibly ceased there are no longer any brainstem reflexes that can occur. Suggestions otherwise have conflated irreversible loss of circulation with permanent loss of circulation in the organism. Very early during the so-called permanent loss of circulation, there may be some brainstem reflexes. However, when absent circulation is irreversible, there are not brainstem reflexes present [77].

Third, some of the confusion underlying this claim is the assertion that ‘permanent’ is a construal of ‘irreversible’ when applied to the concept of death, such that death can be declared using the criterion of permanent loss of circulation (i.e., after 5 minutes when resuscitation is not going to be attempted) prior to irreversible loss of circulation. To fully engage this argument is beyond the scope of this paper; however, we have, in our view, definitively refuted this claim with several arguments published elsewhere [69, 70]. Briefly, there are several reasons why permanent loss of circulation is not a criterion for biological death. One, “death is ordinarily considered to be *irreversible*: no mortal can return from being dead; resuscitation by human action interrupts the process of dying and is not a supernatural resurrection from the state of death” [70]. Two, “treating death as a legal or moral concept that relies on human action or intent [i.e., as permanent] has unacceptable implications”; for example, if “absent circulation in [heart attack victim] Joe was permanent, Joe was already dead at the moment of loss of circulation, and [his rival] Fred’s failure to intervene was not wrong, for he had no obligation to resuscitate a corpse” [70]. Three, death is a state of a body, and patients in the identical physiological biological ontological state, at exactly the same time, should not be considered dead or alive depending on whether resuscitation will be attempted [69, 70]. Four, the capacity (i.e., biological potential) for consciousness is not lost at the onset of permanent cessation of circulation; rather, it is lost when cessation of circulation has become irreversible [70]. For these reasons, permanent loss of circulation is not a criterion for the state of death. Nevertheless, irreversible loss of circulation is a criterion for the state of death, indicating the irreversible cessation of functioning of the organism as a whole.

Another objection may be that exactly when loss of circulation has become irreversible is unclear. In the context of donation after circulatory death “the important question is, at what point after untreated cardiac arrest is the absent circulation known to be irreversible using the very best technology” [70]. This is an epistemic question, and does not change the fact that, metaphysically, irreversible loss of circulation is a valid criterion for biological death.

### Metaphysical conclusion

“We must take the science of death and dying seriously”: the criterion of BD does not meet any acceptable concept of death [36]. In addition, we should “not obscure critically important and fundamentally normative evaluations under misleading language” [23]. We propose that the reason that BD has been accepted is because the patient now may not have any remaining interests or rights in continued existence, may not be capable of being harmed, and may have lost moral status. But we should acknowledge this as the non-scientific, non-factual, moral judgement that it is [23]. The BD organism is not biologically dead.

## The Epistemic Problems (Table 3)

In this section we ignore the metaphysical problem, and are concerned with whether we can know that BD has occurred in an individual patient. We consider two epistemic problems, and consider the second to be insurmountable.

**Table 3:** The Epistemic Problems with brain death, and outline of objections with their main replies

Argument	Objections	Replies
Argument 1: Bedside tests do not confirm the loss of all brain functions: ongoing EEG, EP, and hypothalamic functions, stress responses, breathing at higher PaCO2, brainstem reflexes (often incorrectly labelled as ‘spinal’)	BD has withstood the test of time	-Circular: BD inevitably leads to withdrawal of life support. -False: cases of reversibility are reported (see Table 4) -Wrong question: not about the prognosis of BD, but about whether BD is death
	Residual functions are not critical or clinical	-Ad hoc: why are pupillary reaction and corneal reflexes critical (reflecting ongoing integrative unity of the organism), while EEG and EP functions, neuroendocrine control, and breathing at PaCO2 well over 60mmHg are not? -Circular: “critical functions are necessary for life, and death is the loss of critical functions” -False: neuroendocrine control is a clinical function (just observe urine output) -Self-defeating: only an argument for higher BD [the only function that cannot be replaced mechanically is consciousness]
Argument 2: bedside tests cannot diagnose the loss of all brain functions due to: confounders in all or an unknown number of cases [spinal cord injury during brain herniation; possible total locked-in syndrome; central thyroid and adrenal insufficiency; possible global ischemic penumbra; vaguely described other confounders]; apnea testing being contraindicated, self-fulfilling, and not fit for purpose [does not diagnose loss of medullary function]	Similar to BD, other diagnoses are made according to clinical judgment	-Not appropriate for the diagnosis of death: a final irreversible state with implications that leave no room for error
	An ancillary test can confirm the diagnosis of BD	-EEG: only tests for superficial cortical function -Radionuclide CBF test: poorly studied in terms of specificity for diagnosis of BD versus other severe brain/brainstem injuries. Cases reported of absent CBF with retained brain functions [including EEG, posturing, head-turning, and breathing]. -Unknown prevalence of global ischemic penumbra: Jahi McMath had absent CBF, but lack of brain destruction on MRI and may have emerged to the minimally conscious state.
	Similar to epistemic claims about methods to diagnose death by circulatory criteria	-Tests to diagnose circulatory death are not debated; rather, when the irreversibility of circulatory death occurs is debated

*BD* brain death, *CBF* cerebral blood flow, *EEG* electroencephalogram, *EP* evoked potential, *MRI* magnetic resonance imaging

### Epistemic Problem 1: Do the bedside tests for BD confirm that there has been irreversible loss of all brain functions?

The simple answer is ‘no’. For example, in properly diagnosed cases of BD, the following brain functions continue if tested for: EEG activity in ~20%, brainstem auditory and/or somatosensory evoked potential activity in ~5%, hypothalamic functions (e.g., ongoing antidiuretic hormone regulation of fluid and electrolytes, ongoing temperature regulation, absence of hemodynamic instability) in at least 50%, hemodynamic and endocrine stress response to incision for organ procurement (e.g., rise in heart rate and blood pressure), and the ability to breath at a PaCO2 well over 60 mmHg (several case reports) [6, 78–89]. Other functions described in case reports include the Cushing reflex with bradycardia and hypertension (a vasomotor brainstem function), the oculo-cardiac reflex, and lacrimation [90]. The explanation for these findings may be that, in properly diagnosed cases of BD, the following findings occur if tested for: ongoing cerebral blood flow by radionuclide angiography in ~20%, and lack of extensive brain pathological destruction in over 20% of cases [26, 78, 90–94]. The tests for BD diagnose a neurologically devastating state with a dismal prognosis, but they do not fulfill the criterion of BD itself.

So-called spinal reflexes are present in 11-44% of BD cases [95–99]. For example, the Lazarus sign is present in ~4% of patients with BD: bilateral arm flexion, shoulder adduction, hand raising to the chest or neck, elicited by sternal stimuli, head or neck flexion, or the apnea test [97]. The action can look as if the patient is trying to move the endotracheal tube. Surprisingly, even ‘decerebrate-like’ posturing, ‘head turning’, and withdrawal (flexion movements) to supraclavicular stimulus have been reported in the literature as spinal reflexes in BD [100–106]. These complex polysynaptic reflexes have been attributed to the spinal central pattern generators [95–99]. That these complex reflexes are of brainstem and not of spinal origin is suggested by many important considerations. First, such complex reflexes have never been described in the setting of acute or complete spinal cord injury in either non-human animals or humans [107–112]. Second, acute spinal shock lasts for days or weeks such that the spinal central pattern generators are not active during the period following acute spinal cord injury in both non-human animals and humans [108–119]. Third, when spinal central pattern generators are active they cause rhythmic actions rather than sustained single actions, even in the arms, in both non-human animals and humans [120–128]. Fourth, when spinal mechanisms alone are responsible for these rhythmic activities the muscle movements and electromyography amplitude are very small in both non-human animals and humans [111, 112, 115, 118, 121, 122, 129–131]. Fifth, spinal automatisms, including those of ‘walking movements’, do not occur in primates (including humans) with complete cord injuries without some cord stimulation pharmacologically or electrically to replace the normal tonic supraspinal (i.e., brainstem) input to the spinal central pattern generators [132–142]. Even in humans with incomplete spinal cord injury with some supraspinal input, training with load bearing and manually assisted movements are required to manifest the possible activity of the spinal central pattern generators [124, 137–151]. These movements are in fact difficult to stimulate even in non-primate animals, particularly for the forelimbs [121, 124, 127, 129, 130]. Sixth, brainstem mechanisms, including those from the medullary reticular formation, exert initiation and modulation control over the spinal mechanisms of central pattern generators [152–159]. This supraspinal input is particularly important for flexor muscle activity, and arm movement coupling [149, 157]. Interestingly, when humans with incomplete chronic spinal cord injury have involuntary rhythmic movements it has been noted that these do not occur during sleep, suggesting the need for supraspinal input [160, 161]. This is compatible with incomplete spinal cord injury patients requiring effortful (conscious) contribution to develop stepping movement with training [141, 142]. All of this suggests that so-called complex spinal reflexes in BD are due to clinically observable brainstem activity, as critical as the activity required to blink or gag.

Remaining brain activity should perhaps not be surprising given that the current tests for BD are based on one clinical study of 185 patients that was poorly reported and not prospectively validated, involving critical care capabilities in the 1970s [162]. This Cerebral Survival Study was a prospective multicenter observational study done in 8 intensive care units from 1970-1972. There are over 40 publications of various results from the study, making it difficult to discern some details. Of 616 screened patients, 501 had “deep unresponsive coma and apnea [defined as no effort to override the ventilator for 15 minutes]” [163]. Of these, 316 did not meet BD criteria, of whom 272 (86%) died. Of the 185 who met the retrospective criteria for BD (apnea as defined above, coma, electrocerebral silence, and no known drug intoxication), 114 (62%) had withdrawal of life-support at 24 hours for a diagnosis of BD [163, 164]. Of the 71 (38%) not having withdrawal at 24 hours, 53 (75%) had a cardiac death by <2.9 days (in whom autopsy found respirator brain in 27/35 (77%)), and the other 18 (25%) had a cardiac death at between 2.9-7 days [163–165]. When electrocerebral silence was diagnosed, the systolic blood pressure, usually by brachial cuff manometer, was <80mmHg in 55-60% of the patients [165]. Respiratory efforts were seen “in approximately 2%” of patients after withdrawal of life support on the basis of BD [166]. How many of the patients had absent brainstem reflexes is never stated: of the 501 patients in the study, only 102, all from the same center, had enough information on cephalic reflexes to provide data for analysis, of whom 63 had BD; and at least 45/185 (24%) diagnosed with BD had “non-dilated pupils” [166]. Pupils in BD are 4-9 mm in size, and usually described as dilated [167, 168]. Another retrospective series from the United Kingdom of 609 patients with BD from 1962-1974 found none survived; however, 56% had life-support withdrawn, with median time on ventilation prior to withdrawal or cardiac arrest being 30-40 hours, and >72 hours in only 14% [169].

### Objections

#### BD has withstood the test of time

This is the claim that no cases of reversibility have been reported, thus validating the diagnostic tests. This is problematic for several reasons. First, this is a circular argument. Since the diagnosis of BD invariably leads to withdrawal of, or at least limitation of life-support, it is a self-fulfilling claim that no cases of reversibility have occurred. Second, the claim is false. There have been several cases reported of some reversible brainstem findings of BD [88, 170–176]. This includes cases that have had an apnea test and electrocerebral silence, in both adults (n=2) and children (n=4) [88, 171–175]. Although repeatedly claimed to have been inaccurately diagnosed [including by the American Academy of Neurology], [18, 22, 177] the case reports suggest otherwise (see details in Table 4), as they were diagnosed according to testing compatible with current guidelines. Five of the seven cases were reported after the American Academy of Neurology 1995 guideline (whose details for testing for BD were again confirmed in the 2010 and 2018 updates) [22, 168]. Cases of late recovery of brainstem function after cardiac arrest, especially in those treated with therapeutic hypothermia, should also give one pause about claiming irreversibility in the first several days after brain injury [178, 179]. Third, even if irreversible, the question is not of the prognosis for these patients, but rather whether they had lost all functions of the brain.

**Table 4:** Published cases of reversibility of at least one absent brainstem function occurring after the diagnosis of brain death

Reference	Age	Apnea test done	EEG result	CBF result	Brain Imaging result	Details of the case
Kohrman et al, 1990 [171]	3 months	Yes	ECS	Present on day 6, after the BD diagnosis	Diffuse cerebral edema	Found apneic in crib, requiring CPR. On day 4 and 24 hours later fulfilled BD criteria. Four hours after the second exam developed sucking movements. Over the next 3 days regained eye opening, facial grimacing, eye movements, and corneal reflexes for 30 days until death.
Haun et al, 1991 [88]	3 months	Yes	ECS	Not done	Normal on presentation	Found unresponsive between bed and wall requiring CPR. On day 4 and 24 hours later fulfilled BD criteria. Eight minutes after extubation had spontaneous regular breathing.
Okamato et al, 1995 [172]	3 months	Yes	ECS	Present on day 19, after the BD diagnosis	Severe atrophy on day 19	Hypoglycemia and apnea requiring CPR. On day 3 and 5 fulfilled BD criteria. Regained spontaneous respirations on day 43 until death on day 71.
Shewmon, 2018 [173]	2 years	Stopped at PaCO2 58 mmHg due to desaturation	Likely ECS	CPP zero for several hours. Absent DPTA flow.	Massive cerebral edema with herniation	Severe TBI. Diagnosed BD at 72 hours: met all criteria for BD (ancillary CBF test done because apnea test could not be completed). After extubation had spontaneous breathing.
Roberts et al, 2010 [174]	26 years	Yes	Not done	Present on MRI in MCA, after the BD diagnosis	Uncal and tonsillar herniation with generalized cerebral edema	Mastoiditis and temporal lobe abscess, with normal otoscopy. At 7 hours fulfilled BD criteria. In operating room at 28 hours regained spontaneous breathing.
Webb et al, 2011 [175]	55 years	Yes	ECS	Absent at hour 200, after the BD diagnosis	Diffuse cerebral edema	Cardiac arrest, cooled to <35 degrees for up to 10 hours until hour 55. At hour 72 and 78 met all criteria for BD. In operating room at hour 98 had cough, corneal reflexes, and spontaneous breathing.
Latorre et al, 2020 [176]	59 years	No, due to hemodynamic instability	Not done	Absent on SPECT	Intracerebral hemorrhage, cerebral edema, transtentorial and tonsillar brain herniation	Catastrophic spontaneous intracerebral hemorrhage. Clinical exam at 51 hours compatible with BD, but no apnea test done. Tc-99m Bicisate SPECT scan at 52 hours confirmed absent uptake. “The following morning” he had “cough, intermittent spontaneous respirations, and extensor posturing of the right arm and leg to noxious stimulation.”

*BD* brain death, *CBF* cerebral blood flow, *CPP* cerebral perfusion pressure, *CPR* cardiopulmonary resuscitation, *ECS* electrocerebral silence, *EEG* electroencephalogram, *MCA* middle cerebral artery, *TBI* traumatic brain injury

#### The residual functions are not critical or clinical functions

This claim has been made in different forms. One is that the residual functions detected are mere activities of nests of cells, and not functions [17]. Before developing the claimed distinction between function and ‘mere activity’, the claim was stated in other ways, saying that the residual functions are either insignificant, not clinical functions [and BD is a clinical diagnosis], or not critical [because they are replaceable mechanically] [14, 15, 17, 78]. All of these claims fail for several reasons. First, the claims are ad-hoc, stated without a clear reason other than to save the BD tests. For example, why are pupillary and corneal reflexes significant functions, reflecting integration of the organism as a whole, while EEG activity, brainstem evoked potential activity, neuroendocrine control, and breathing at PaC02 well above 60 mmHg are not? How to define critical, and why these must be clinical functions is not explained, except for the circular claim that critical functions are necessary for maintenance of life, and death is the loss of critical functions [36, 180]. In addition, the clinical versus nonclinical distinction is irrelevant, as a neurologists’ epistemic access to a function is not a relevant consideration to diagnosis of a critical function [23, 181]. Second, the claims are false. Neuroendocrine control can be diagnosed at the bedside by observing lack of polyuria. The spatial resolution of EEG suggests there is widespread neuronal activity when EEG activity is detected, potentially performing functions. Evoked potential activity is due to transduction of ambient energy into electrochemical signals conducted to the brain, suggestive of a function. Neuroendocrine control maintains free water homeostasis, suggestive of a function. Third, the claims are self-defeating [23, 181]. Since breathing can be replaced mechanically it apparently is not a critical brain function. Since only consciousness cannot be replaced mechanically, this is only an argument for higher brain death, and not for the biological death of an organism. Finally, specifically regarding neuroendocrine function, some have claimed that since parts of the hypothalamus and pituitary receive blood flow from the inferior hypophyseal arteries that branch off the extradural segments of the internal carotids, it can be expected that neuroendocrine function may remain [79]. There are problems with this claim. For one, even if true, the fact remains that over half of patients currently diagnosed with BD have this remaining brain function, and should not be declared to have BD. In addition, the origin of blood flow that must reach the intracranial contents seems irrelevant to the claimed pathophysiology of BD: this blood flow would be as restricted as intracranial blood flow from other sources (e.g., internal carotid arteries) by the presumed very high intracranial pressure. It is more likely that the hypothalamic function reflects an area of the brain more resistant to low intracranial blood flow than other parts of the brain [182].

### Epistemic Problem 2: Can the bedside tests for BD confirm that there has been irreversible loss of all brain functions?

The simple answer is ‘no’. This requires detailed explanation, and there are several reasons for this epistemic problem.

First, there are currently unacknowledged confounders to the examination for BD in virtually all cases. A confounder is a condition that may interfere with the ability to do any of the tests for BD, and this is different from a mimic of BD (a mimic can account for all of the tests for BD) [183, 184]. Either a confounder or a mimic precludes the ability to make a clinical diagnosis of BD [183, 184]. One confounder is high cervical spinal cord injury that, although not by itself capable of causing all of the findings of BD, does interfere with the ability to test for response to noxious stimuli [e.g., withdrawal, or posturing responses] and for apnea. Brain herniation, the usual pathway leading to BD, causes potentially reversible cervical spinal cord injury in most cases, by causing direct compression of the cervical spinal cord, or the anterior spinal arteries, causing ischemic injury to the cord [185]. It is important to point out that this spinal cord injury from brain herniation has often been reported to be reversible (including reversal of apnea and paralysis), and thus cannot be confirmatory of complete brain destruction [185]. Even if one ignores the potential reversibility of the injury, the fact remains that spinal cord injury is a confounder to the clinical examination for BD. There are only two pathological series of BD that examined the spinal cord, finding in 56-100% of cases upper cervical spinal cord damage [92, 164, 186]. It would be impractical to have MRI for every case of suspected BD to verify whether spinal cord damage has occurred, and the accuracy for this indication would require study. A second confounder is primary brainstem (infratentorial) injury that can lead to all [a mimic] or some [a confounder] of the clinical findings of BD, without fulfilling the irreversible loss of consciousness requirement [26, 187, 188]. If the brainstem is injured to the point of a total locked-in syndrome, which theoretically can occur if the meso-pontine tegmental reticular formation is relatively spared [e.g., from basilar artery thrombosis, brainstem or cerebellar bleed, subarachnoid hemorrhage], this can lead to all the clinical findings of BD [i.e., so-called ‘brainstem death’] but with preserved capacity for consciousness [187]. In this situation, after allowing an unknown sufficient time for recovery from brainstem edema, one would expect the EEG to show alpha or alpha/theta activity, indicating preserved function and connectivity of the meso-pontine tegmentum [187]. How often this may occur is unknown, but primary brainstem injury leading to a mimic of BD [i.e., total locked-in syndrome] may occur in as many as 10% of cases of primary brainstem injury leading to the findings of BD [26, 187]. To rule out this confounder/mimic, it has been suggested that an EEG and/or cerebral blood flow test is required to confirm lack of EEG activity or lack of brain blood flow [187]. In Canada, it has been argued that EEG only detects cortical activity and is subject to artifact in the ICU setting, and thus is not considered an ancillary test [167]. This only worsens the epistemic problem, and does not negate the need for an EEG to confirm lack of meso-pontine tegmental function when infratentorial injury is a cause of the BD syndrome. A third confounder is adrenal or thyroid deficiency that also often accompany brain herniation. The pituitary or hypothalamus should be and often are damaged if there is loss of all brain functions, and this would result in secondary adrenal or thyroid deficiency [79]. These endocrine abnormalities are known to cause poor respiratory response to PaC02 and coma, and are therefore recognized confounders to the examination for BD [189]. How to diagnose and treat these in the setting of suspected BD is unclear, and would require study. Yet another confounder present in an unknown number of cases is the global ischemic penumbra (GIP), to be described later [190].

Second, acknowledged confounders to any part of the examination for BD are vaguely described and left up to variable and inconsistent clinical judgement. There are many examples of this problem. Unresuscitated shock: what blood pressure and measure of tissue perfusion is adequate? Hypoxia: what partial pressure of oxygen is adequate? Metabolic disorders: what ones, and what exact level of acidosis or hyperammonemia? Biochemical abnormalities: what exact level of glucose, sodium, calcium, magnesium, phosphate, liver function, and renal function? Peripheral nerve or muscle dysfunction: how should this be diagnosed, of what severity, and does critical illness polyneuropathy or myopathy count and thus require exclusion? Clinically significant drug intoxications: which drugs, and at what exact measured level? Brainstem encephalitis: how should this be diagnosed, and how can reversibility be determined?

Third, the apnea test is both contraindicated and poorly suited for purpose [i.e., the purpose of determining loss of medullary function] [189, 190]. The test is contraindicated because, in the setting of a raised intracranial pressure, a rise in PaCO2 can be expected to increase this pressure further, thus reducing cerebral perfusion and resulting in no-reflow phenomenon [189–194]. This can convert ischemic penumbra tissue to irreversibly injured brain [190]. Jastremski et al commented that they “have observed dramatic increases in intracranial pressure when PaCO2 was allowed to rise to normal levels for an apnea test” [191]. Thus, the test itself creates a self-fulfilling prophecy, by causing apnea to occur due to the increased or completed herniation resulting from the test itself. The test is poorly suited for purpose because it does not test for medullary function, the stated reason for doing the apnea test [189]. In the setting of a medulla isolated from the pons, gasping is the respiratory pattern reproducibly obtained, and this is induced by hypoxia, prevented by hyperoxia, and not induced by hypercarbia [189, 195–197]. When the preBotzinger complex in the medulla is damaged, hyperoxia causes fatal apneas [189, 198]. Yet, the apnea test induces hyperoxia and hypercarbia, and thus does not test for medullary respiratory function at all, and can induce the apnea it tests for. Testing isolated medullary function would require inducing significant hypoxia, and would not be acceptable.

### Objections

#### Similar to BD, other diagnoses are made according to clinical judgment

This may be true for some other diagnoses. However, for a diagnosis of death, it would seem that patients expect that if one is diagnosed dead in one place he/she should also be diagnosed as dead in another place. Death is a final state, and leaves no room for error. People over time have feared false negative diagnoses of death, and the reliability of the indicators used by physicians in diagnosing death were what was of concern [199]. The question for medical science has always been - is there a reliable means to determine the irreversible state of death? It is doubtful that patients would accept that variable and inconsistent clinician judgement is what determines whether they are in fact dead.

#### An ancillary test can confirm the diagnosis of BD

In the United States an isoelectric EEG is considered confirmatory of BD [200]. Of course, this is a flawed argument, because an isoelectric EEG indicates absent superficial cortical function, but does not test for any of the brainstem functions thought so critical to the diagnosis of BD [167].

In most countries, lack of cerebral blood flow on radionuclide brain perfusion is considered confirmatory of BD [167, 168, 200–202]. There are several problems with this reliance on perfusion scans. First, in the only study of the specificity of non-diffusible radionuclide angiography, the specificity was 56%, with 5/9 patients without clinical BD having absent flow [203]. Second, for the (likely more accurate) diffusible radionuclide tracers a recent review found that all studies suffered from a referral bias, most data came from one study (n=22), and specificity was 41/41 (100%, 95% CI 92.6 to 100%) [91]. For SPECT imaging, studies had the same referral bias, and specificity was 12/12 (100%, 95% CI 78.4 to 100%) [91]. The tests have not been subjected to rigorous testing to determine specificity in patients with severe brain injury and brainstem involvement but without fulfilling complete criteria for BD. This is important because the tests for brain blood flow are being used not to give a prognosis of poor neurological outcome or death, rather, to diagnose the state of death. Supporting the concern regarding specificity are cases of retained brain functions and absent cerebral perfusion [e.g., decerebrate posturing, head turning from side to side, neuroendocrine function], and of retained EEG activity and absent cerebral perfusion [90, 91]. In one reported case a 54 year old male after a prolonged cardiac arrest had absent radionuclide brain perfusion using hexamethylpropylene-amine oxime but spontaneous respirations “several hours later” [pg. 131, Figure 4] [204]. In another case a 59 year old after intracerebral hemorrhage had absent radionuclide perfusion using Tc-99m Bicisate SPECT scan but “the following morning” had “cough, intermittent spontaneous respirations, and extensor posturing of the right arm and leg to noxious stimulation” [176]. These cases suggest that flow to some (critical) areas of the brain below the detection limit of the radionuclide test continued, likely in the penumbral range, with function later emerging when blood flow presumably increased. In fact, blood flow being in the penumbral range at some point is a logical necessity in the pathophysiology of BD; blood flow must fall continuously as intracranial pressure rises (and cerebral perfusion pressure falls), passing through penumbral ranges until reaching the critical closing pressure of brain vessels [190].

The recent case of Jahi McMath also suggests that lack of cerebral blood flow on testing may reflect a GIP, meaning, blood flow high enough to prevent brain tissue necrosis but low enough to produce a loss of clinically detectable brain function [32, 205]. This GIP may have resulted in lack of detectable brain functions and perfusion that only produced a mimic of BD. We base this possibility on two considerations. First, Jahi’s brain blood flow was below the detection limit of the test [a diffusible radionuclide cerebral blood flow perfusion test], yet later [as Shewmon has hypothesized, based on video evidence provided by the family] “intermittent increases in cerebral blood flow above the penumbra range permitted cerebral function to return intermittently [to a minimally conscious state], manifested by responsiveness to commands” [205]. Admittedly, this is controversial as the video evidence has been considered unscientific and unreliable by many [32]. Nevertheless, Shewmon reported that “6 days before she died, I visited her in her hospital room and observed a (non-myoclonic) right arm movement in response to her mother’s command to move that arm. (There had been no spontaneous movements of any kind up that point or for the rest of my visit, so it was clearly not a chance coincidence of a random baseline movement)” [32]. Second, even if one were to disregard the video evidence as unreliable, the structural preservation of most of Jahi’s brain on MRI done over 9 months after her BD diagnosis must suggest there has been sufficient blood flow to prevent cell death despite her absent flow on the nuclear medicine test. Shewmon argued that “there is no other possible explanation, except to dogmatically disregard the gross structural preservation [of brain] on MRI” [205]. Thus, we argue that GIP may be another confounder for the examination for BD, present in an unknown number of cases, and at present beyond our ability to diagnose with certainty.

#### Similar epistemic claims could be made about methods to diagnose death using circulatory criteria

The objection is that there is debate about the tests to be used to diagnose death using circulatory criteria, and an operational definition was arrived at in that case. This claim is false. The tests used to diagnose the circulatory criterion of death are not debated: absent circulation can be verified by stethoscope, electrocardiogram, or arterial line, supplemented by palpation. There is not a problem of confounders always being present that preclude epistemic access to whether absent circulation has occurred. The debate is about when loss of circulation becomes irreversible, not about the tests used to diagnose death [70]. As mentioned above, we have, in our view, definitively refuted the claim that ‘permanent’ is a construal of ‘irreversible’ when applied to the concept of death with several arguments published elsewhere [69, 70]. The objection might be that precisely when absent circulation has become irreversible is not known. Although this problem also applies to BD [i.e., when the findings of BD become irreversible], the problem is different from the epistemic problems with BD discussed above. In addition, we can be highly certain that after 1 hour of untreated cardiac arrest effective circulation sufficient to enable the integrative unity of the organism as a whole cannot be restored [69]. We also know that loss of circulation for 5, 10, or even 20 minutes is still potentially reversible.

### Epistemic conclusion

As currently diagnosed, BD cannot be said to be the state with lack of all brain functions. Moreover, BD is not currently possible to diagnose [i.e., the irreversible lack of tested clinical brain functions] due to confounders. In addition, the apnea test should be abandoned because it does not test isolated medullary function and is a self-fulfilling test. These problems do not change the fact that BD, as currently diagnosed, is a devastating neurological state where regaining sentience is very unlikely.

## Potential ways forward (Table 5)

We have argued that BD is not the biological death of the human organism, and even if it was, we cannot make the diagnosis accurately in practice. We believe that this leaves several options for moving forward in what to do about the diagnosis of BD. Here we discuss some difficult issues with each proposal, and conclude with what we believe is the best option. Our purpose is not to abandon the diagnosis of BD, but rather to attempt to preserve current practice after a patient is diagnosed as having BD according to future guidelines improved to address some of the epistemic problems we raised. Current practice is to allow one of two options: vital organ donation, or unilateral withdrawal of life-support. We do not consider the option of ignoring all of these problems, as we believe the issues will not go away, and we must face them honestly.

**Table 5:** Potential ways forward that accept both the intractable metaphysical and epistemic problems with brain death

Potential solution	Pro	Con	Conclusion
Accept higher BD: death of the person	-Likely correct: compatible with the transplant intuition, the remnant person problem, and considerations of conjoined twinning	-Unacceptable implications for some: religions [is the human person separable from the human organism], and society [irreversible PVS, early fetus, and possibly neonates are not alive, and thus have no rights]	Likely not acceptable
Accept BD as a legal fiction	-Treat the BD as analogous to the dead in law, as they lack an interest in continued existence	-Legal fictions are known to be fictions: would need to acknowledge that BD is not really death -The reason for the analogy would also apply to higher BD, raising the problems above	Likely not acceptable
Abandon the dead donor rule	-Acknowledges the metaphysical and epistemic problems -Respects non-maleficence (duty to do no harm) and autonomy (duty to obtain informed consent) -Maintains trust in medicine by being trustworthy	-Need to acknowledge the analogy to withdrawal of life-support as a form of justified killing, and thus not murder -Possible adverse effect on organ donation rates and trust in organ donation (and medicine)	Likely acceptable

*BD* brain death, *PVS* permanent vegetative state

### Accept the higher brain death criterion of death of the person

This would mean accepting the implications of a personhood based concept of death, namely, that there are two deaths [of *me*, the human person; and, of my organism], and that the early fetus, anencephalic newborn/infant, and the patient with irreversible PVS from any cause, are not living human persons (i.e., not one of *us*, not essentially the same as *me*), but rather are human organisms [37, 47–51]. Certain behaviors could be tied to each of the two deaths [206]. With higher brain death, vital organ donation, withdrawal of biological life-support, and mourning would be appropriate. With biological death of the organism, burial, cremation, and autopsy would be appropriate. There would be required changes to the legal system, including that murder apply only to wrongfully causing the death of the person, and thus organ donation from the living organism of a dead human person could not be considered murder. We are sympathetic to this view, and consider the remnant-person argument and considerations about conjoined twins discussed above to prove that it is true; indeed, this is likely why (unknowingly) BD has been ‘accepted’ by society to date.

Nevertheless, this would be a ‘hard-sell’ to the public and legislators for several reasons. First, some religions may not accept that the human person is separable from the human organism, or that a human organism is not owed the same respect and consideration as the human person [23]. For example, there is debate in Catholicism about when the so-called rational hylomorphic soul may leave the biologically living human organism [34, 207, 208]. Controversy is likely, given the similar controversies regarding abortion [68]. Second, allowing personal choice in the matter of whether to accept two deaths might be a solution to the first problem, but may be too complex to implement in practice. Third, this raises an epistemic problem again: how to diagnose irreversible loss of the capacity for consciousness. We currently have no highly specific way to diagnose irreversible loss of consciousness, as shown by recent reports of late recoveries from persistent vegetative state and of cognitive motor dissociation states [209, 210]. The state of BD as currently diagnosed may (or may not- see above for discussion of the case of Jahi McMath) indicate this; however, in principle, this state will occur in many patients not fulfilling criteria for BD. It seems odd to have patients in an irreversible PVS being cared for by family and health-care workers, even being subjected to rehabilitation treatments, when it turns out they were already dead long ago. The epistemic problem is not just how will we know how to make the diagnosis, but also how will we cope with having stood by the patient for so long when we discover they were actually dead years ago. Fourth, some argue that not just consciousness, but self-aware consciousness is required for personhood, to be one of *us* [37, 49, 51]. For example, what if you remained sentient, but lost all memories and personality traits due to brain disease, and then medical science discovered how to restore brain functions so that you would from that time forward develop new memories and personality. Will this person, with no psychological connectedness or continuity with your previous psychological states, still be you? Some argue that this would be a new person altogether. This would imply that many more cases of higher brain death exist than just those in irreversible PVS, and also that newborns are not yet one of *us*. This is unlikely to be acceptable to society.

### Consider BD being equivalent to death as a legal fiction

This proposal is to consider BD as a status legal fiction, an untruth that is treated as true by the law in the service of particular legal ends [27, 28]. For example, the law treats corporations as persons in order to extend a well-developed body of law to corporations, or the law treats certain war veterans with particular diseases as presumed to have become ill during service, so they are eligible for free treatment. Similarly, the law could treat the BD as analogous to the actually dead because they are relevantly similar for determining what law should apply to them [27, 28]. In the state of BD, and arguably despite the epistemic problems, no harm is done in allowing the patient to die, even by organ donation, as the patient is no longer sentient, and thus no longer has an interest in continuing such an existence.

Again, there are several problems with this proposal. First, legal fictions are known to be fictions [27, 28]. To treat BD as a legal fiction would require that the public is aware that the patient is not really biologically dead, but can be treated as such under the law [27, 28, 211]. At present, both the public and medical professionals are not informed that this is a legal fiction, and this would need to change. Second, the reason for the analogy between the BD and the actually dead would also apply to those who are not BD but have irreversibly lost consciousness, raising similar problems to those of accepting a higher brain death concept. Third, this proposal has been meant to allow current practice to continue while working towards better changes to the law that do not consider organ donation from these patients as murder [27, 28]. According to this argument, the problems with accepting a higher brain death concept will again occur.

### Abandon the dead donor rule

The main reasons for accepting BD as death have been to free up scarce intensive care beds and resources, and to allow vital organ donation without the procurement killing the patient [181, 212–215]. When a patient is BD, the current options are to withdraw intensive care interventions (as they are no longer useful in a corpse), or to allow vital organ donation prior to the withdrawal of intensive care interventions (these interventions being temporarily used to sustain the organs). The dead donor rule argues that prior to vital organ donation the donor must be dead, as otherwise the act of organ procurement is what kills the patient [26, 213–216].

Considering BD to be death is no longer necessary in order to withdraw life-support. BD is a devastating neurological injury that is likely not compatible with regaining sentience in the vast majority of cases. In the vast majority of cases, the patient likely no longer has interests that matter to them, and the person will never regain consciousness. To reiterate, in the state of BD the chance of ever regaining sentience is approaching zero, as among the cases we review of reversal of any signs of BD [Table 4], only Kohrman’s case [171] and possibly Jahi McMath [205] may plausibly be claimed to have regained any minimal degree of sentience. Family can be counseled that it is time to withdraw life-support and allow the patient to die. Society might even decide that withdrawal of life-support is mandatory in this situation given distributive justice concerns. Some might worry that a family could refuse consent to withdrawal of life-support; nevertheless, we fail to see how obtaining consent to withdrawal of life-support from a biologically living patient with BD would be more problematic than obtaining consent to withdrawal of life-support from an equally non-sentient biologically living patient with the only brainstem functions of some respirations or an isolated corneal response [i.e., an equally dismal prognosis].

For similar reasons, society could decide that vital organ donation in the state of BD, with prior patient or family consent, although killing, would not be considered murder. This is already the case when life-support is withdrawn from severely neurologically damaged patients [217, 218]. During withdrawal of life-support in a patient who cannot breathe or sustain circulation effectively, the act of withdrawal (of, say, the ventilator, or the vasopressors, or the extracorporeal circulatory support) directly results in the death of the patient when circulation irreversibly ceases. The act of withdrawal is thus the proximate cause of the death occurring when it does. In the metaphysics of causation, according to Mackie, the act [withdrawal] is a non-redundant member of a minimal sufficient condition for the effect [death]; therefore, the act is an “insufficient but necessary part of a condition which is itself unnecessary but sufficient” for the death (i.e., a causal INUS condition) [219]. According to Lewis’ counterfactual theory of causation, if withdrawal does not occur, then death does not occur, such that death depends counterfactually and causally on withdrawal. More abstractly, in Lewis’ possible worlds terminology, worlds with withdrawal where death holds are closer to our actual world than is any withdrawal world where death does not hold [220]. This causing of death is killing, but is justified by prior decisions that this is in the best interests of the patient, with prior consent of the patient or decision-maker [217, 218]. An objection would claim that this is not killing, but is simply allowing nature to take its course, thus allowing a natural death; this attributes death to the disease, not to the physician’s act. This objection fails. First, withdrawal is an act, not an omission [i.e., not an allowing]. Second, consider this example. If prior to a decision to withdraw life-support an angry family member or healthcare worker were to barge in and extubate the patient, saying that ongoing ventilation was disrespectful to the patient, and the patient then died, that act would be considered murder, and the cause of death attributed to the family member’s or healthcare worker’s action. This is because the killing would be considered not yet justified. When the identical action has the identical consequence (i.e., the same causal sequence) but follows discussion that justifies the plan by agreement that ongoing ventilation is disrespectful to the patient, this is not murder – it is justified killing [217, 218]. Similarly, if prior informed consent is obtained, procurement of vital organs from a BD patient would be considered justified killing, and not murder [217, 218]. This would require a change in current legislation to recognize this exception to laws about killing [213, 214, 216–218]. Instead of blind obedience to the dead donor rule, this would prioritize and improve compliance with the principles of non-maleficence [duty to do no harm] and respect for autonomy [duty to obtain informed consent] [181, 213, 217, 218]. Again, we emphasize that in the state of BD the chance of ever regaining sentience is approaching zero, and thus the biologically living organism no longer has an interest in continuing such an existence.

Some may still not agree with our use of the term ‘killing’. We have, with Truog et al, assumed ‘killing’ and ‘causing death’ are equivalent [217, 218]. We have also, with Nair-Collins and Truog et al, attempted to distinguish ‘causing death’ and ‘wrongfully causing death’ [36, 181, 217, 218]. Sulmasy proposed different terminology, reserving morally proscribed ‘killing*’ [the asterisk indicates a neologism] for causing death by an action that introduces a new lethal pathophysiological state and with the intention that the patient should die by way of one’s act. He uses ‘allowing to die*’ [another neologism] to indicate causing death by an action that removes an intervention that forestalls or ameliorates a pre-existing fatal condition; this is morally proscribed if the intention is that the patient should die by way of one’s act, and morally permissible if the intention is only to abate treatment that is too burdensome for the patient [216, 221, 222]. Sulmasy suggests a test to determine intention: ask “How would one feel and what would one do if the patient were not to die after one’s action?”; if one would “feel that one had failed” or one would “try to figure out how to finish her off”, then “the patient’s death was probably intended” [221]. We are not convinced about ‘allowing to die*’ terminology. First, regardless of terminology used, withdrawal of life-support is active [i.e., not an omission] and causal [i.e., nature is not what causes the death] of death [221]. Whether one calls the causal action ‘killing’ or ‘allowing to die*’, it is morally permissible if justified by other factors. We argue that withdrawal of life-support and vital organ transplantation in the setting of BD are justified by the same moral principles of non-maleficence and respect for autonomy. And both can meet Sulmasy’s test: if the patient did not die one would not feel they had “failed”, nor try to figure out how to “finish her off”. The intent of the action can be said to be to remove treatment or to allow organ donation prior to removing treatment respectively. Second, intentions are difficult to know, even for the agent. We believe that the subtle difference between the intentions is based on rationalization as a post-hoc attempt to justify our immediate non-reflective [‘common-sense’] intuitions that intending death is always wrong [223, 224]. For example, isn’t removing the endotracheal tube in someone who has no capacity to breathe effectively “introducing a new lethal pathophysiological state” that causes the death to occur when it does? And in our imagined cases, can’t each agent claim he intended only to withdraw the ventilator treatment? And would it really have been so wrong to withdraw the ventilator if the physician intended death to occur? And would it be better to put the BD patient on extracorporeal support, then remove the vital organs, and only then remove the extracorporeal support, in order to comply with the somewhat contrived ‘allowing to die*’ definition of merely causing death by an action that removes an intervention [the extracorporeal support] that forestalls or ameliorates a pre-existing fatal condition, aiming only to abate treatment that is too burdensome for the patient? Third, the debate focuses on what makes killing morally wrong. Marquis has argued that a sufficient explanation is that wrongful killing “deprives one of all the experiences, activities, projects, and enjoyments that would otherwise have constituted one’s future”, of a “future of value”, a “future like ours” [225, 226]. DeGrazia has argued that “what matters in survival” is “our continuing existence as persons – beings with the capacity for complex forms of consciousness – with unfolding self-narratives and, if possible, success in self-creation” [227]. It follows that in BD, killing by vital organ donation is not morally wrong.

There are potential problems with abandoning the DDR. First, there is concern about devastating consequences for organ transplantation, as the public might lose trust in the institution of medicine and vital organ donation [228, 229]. Bernat claims that the DDR “is an indispensable ethical protection for dying patients who plan to donate organs and one that strengthens public trust and confidence in our voluntary system of organ donation… Many people harbor a fear that physicians have a greater interest in procuring their organs than in their welfare. They need the reassurance provided by the DDR” [228]. This is an important concern, and it is likely that many will (at least temporarily) lose trust in medicine and vital organ donation, particularly people that struggle with trust at baseline. Even so, we believe this objection fails. We already violate the DDR. As Nair-Collins has pointed out, "biological reality [i.e., biological death] is what it is, whether we like it or not... What the argument [to deny biological reality in order to save organ transplantation from potential adverse effects] advocates, however, is for the medical community to intentionally deceive the public about the biological reality of death" ([23], at 681). He goes on to say that "trust is at the foundation of medicine...[the argument] advocates doing something that is antithetical to the very existence of the institution of medicine..." ([23], at 681). Others have also pointed out that “it is not clear that that [the potential decrease in organ donation] would justify anything other than a piece of large scale public dishonesty" ([230], at 67). We believe that in the long run it is best to maintain trust by being trustworthy, and that this will have the best effects on the practice of medicine, including organ transplantation. Assuming otherwise may underestimate the public’s capacity to understand the condition of patients in BD and why they may not be harmed from vital organ donation with consent. It is interesting that many lay-press presentations of BD organ donors already seem to agree with this - claiming that the patient “is on life-support” and “died after going for organ donation ” [217].

Second, there is concern about a slippery slope. Bernat claims the DDR “protects vulnerable people such as anencephalic infants and incarcerated prisoners” [228]. The worry is that vital organ donation that violates the DDR will be extended to these and other vulnerable patients. We believe this is very unlikely. Here we advocate for violating the DDR in the setting of BD [which is already done, according to our arguments], with voluntary non-coerced informed consent, and we argue this will increase respect for non-maleficence and autonomy in these vulnerable patients with BD. Beyond the scope of this paper, we also advocate for violating the DDR in the setting of donation after circulatory death [which is already done, according to our arguments], with appropriate safeguards [69, 70]. Safeguards would include medical consensus on the dismal prognosis, prior decision to cause death by withdrawal of life-support based on assessment of benefits/burdens, voluntary informed consent, better ways to avoid conflicts of interest, and anesthesia at the time of organ procurement. Again, we believe this will increase respect for non-maleficence and autonomy in these vulnerable patients who are dying and near death. It is interesting to note that these safeguards better protect vulnerable patients than the currently accepted withdrawal of life-support, which can be based upon an individual physician’s assessment of prognosis, and influenced by unconscious cognitive biases [231, 232].

The option of preserving the DDR by abandoning vital organ donation after BD we find much less desirable, as this would not respect the principles of non-maleficence [i.e., would harm many potential organ recipients, without providing any benefit to the potential organ donor], nor autonomy [i.e., would not respect the wishes of many potential organ donors who come to be in the state of BD], which underlie the supposed need for the DDR in the first place [36, 181]. In other words, we do not support abandoning the DDR merely to avoid the inconvenient implications for organ transplantation; rather, we support abandoning the DDR for principled ethical reasons as described above.

Abandoning the dead donor rule in the setting of BD is likely the best course in moving forward [213, 214, 216–218]. This would allow all of us to admit that BD is not biological death, that our current tests cannot confirm that all brain functions are absent, and that the best course of action in BD is withdrawal of life-support with the option of vital organ donation. This might also allow patients having withdrawal of life-support for other reasons, who are almost certain to die after this withdrawal, to consent to the option of vital organ donation prior to withdrawal of life-support [233]. This last possibility requires open public discussion.

## Conclusions

There are two intractable problems in the BD debates. First, the metaphysical problem: there is no reason that withstands critical scrutiny to believe that BD is the state of biological death of the human organism. Second, the epistemic problem: there is no way currently to diagnose the state of BD, the irreversible loss of all brain functions, using clinical tests and ancillary tests, given potential confounders to testing. These problems are intractable in that there has been no solution offered other than bare assertions of an ‘operational definition’ of death. We argue that the best solution is to accept both the metaphysical problem - that BD is not biological death of the human organism- and the epistemic problem - that as currently diagnosed, BD is a devastating neurological state where recovery of sentience is very unlikely, but not a confirmed state of irreversible loss of all [critical] brain functions. We can achieve this solution by abandoning the dead donor rule, thus allowing vital organ donation from patients currently diagnosed as BD, assuming appropriate changes are made to the consent process and to laws about killing. This has the added advantage of allowing the giving of anesthesia during organ procurement from the biologically living organ donor.

## Data Availability

Not applicable

## Abbreviations

BD: Brain death
EEG: Electroencephalogram
GIP: Global ischemic penumbra
